# From digital learning readiness to student satisfaction: the mediating role of learning well-being in Saudi higher education

**DOI:** 10.3389/frai.2026.1859906

**Published:** 2026-08-17

**Authors:** Aliyu Alhaji Abubakar, Essa Mubrik N. Almutairi, Hilal Nafil Alhulail, Sayf Mohamed Dhiya Younis Al-Ashqar, Habiba Muhammad Abdullahi

**Affiliations:** 1 Department of Management Information System, College of Business, University of Hail, Ha'il, Saudi Arabia; 2 Humanities Research Centre, University of Hail, Ha’il, Saudi Arabia; 3 Department of Marketing, College of Business, University of Hail, Ha’il, Saudi Arabia; 4 Business Administration Department, College of Administration and Economics, University of Mosul, Mosul, Iraq; 5 Department of Entrepreneurship & Innovation Studies, Federal University Kashere, Kashere, Nigeria

**Keywords:** digital education, digital learning awareness, higher education institutions, learning well-being, Saudi Arabia, student satisfaction

## Abstract

This research advances understanding of the determinants of student satisfaction in digital higher education, offering valuable insights for sustainable digital transformation initiatives globally. The study sampled 212 actively engaged higher education students from the Hail region of Saudi Arabia, with a gender distribution of 55.2% males and 44.8% females, utilizing a culturally adapted online questionnaire comprising validated scales on digital learning, acceptance, readiness, orchestration, well-being, and satisfaction, measured on a five-point Likert scale. Using TAM, UTAUT, Digital Readiness Theory, and well-being theory, this quantitative study (PLS-SEM) find that digital learning readiness and institutional orchestration directly improve students’ well-being, which in turn drives students learning satisfaction in Saudi higher educations institutions. These findings extend digital learning theory by demonstrating that well-being operates as a full mediator for acceptance, readiness, and orchestration, refining theoretical predictions about boundary condition of TAM and UTAUT in collective educational context. The findings suggest that institutions should strengthen digital strategies by improving infrastructure, developing staff digital competencies, and expanding student mental-health support.

## 1 Introduction

Over the past decade, digital learning has rapidly evolved from a supplementary instructional mode to a central pillar of higher education systems worldwide. The global e-learning market, valued at over USD 399 billion in 2022, is projected to surpass USD 1.8 trillion by 2032, reflecting an annual growth rate of more than 14% (Sharma and Sharma, 2025). Understanding factors shaping students’ satisfaction is essential for ensuring sustainable educational transformation.

The Arab region witnessed a nearly 50% increase in digital learning platform usage (Alduosari et al., 2025). Governments across the Gulf Cooperation Council (GCC) have invested heavily in digital infrastructure, with Saudi Arabia ranking among the top countries in digital readiness. The Kingdom’s ICT spending exceeded USD 70 billion in 2023, making it the largest technology market in the Middle East (Fareed et al., 2025).

In alignment with Vision 2030, Saudi Arabia’s higher education sector is undergoing a profound transformation that emphasizes digital innovation, human capital development, and educational excellence. The Ministry of Education has prioritized digital learning as a core strategy, leading to the establishment of the National eLearning Center (NELC) and the accreditation of more than 1,500 digital courses by 2023 (Al Atawi et al., 2025). Saudi universities such as King Saud University, King Abdulaziz University, and Princess Nourah University have integrated blended and fully online programs at unprecedented scales (Alfawaz and Yamin, 2020). However, the success of these initiatives depends not only on technological availability but also on students’ awareness, acceptance, readiness, and well-being within digital learning environments.

With respect to digital learning awareness, students’ understanding of digital tools plays a foundational role in shaping learning outcomes. Research shows that students with higher awareness of digital learning resources demonstrate greater engagement and performance (Wong et al., 2025). In Saudi Arabia, however, awareness levels vary significantly across institutions and disciplines, particularly between urban and rural higher education institutions (Alghamdi, 2017).

Similarly, acceptance of digital learning technologies is equally pertinent. The Technology Acceptance Model (TAM) posits that perceived usefulness and ease of use strongly influence students’ willingness to engage with digital platforms (Davis, 1989). Studies in the GCC show that over 40% of students express hesitation toward fully online learning due to concerns about interaction quality, assessment fairness, and technological reliability (Fazza and Mahgoub, 2021). Therefore, understanding these dynamics is essential for designing interventions that foster positive attitudes toward digital learning.

Digital readiness encompassing technological skills, self-regulation, and psychological preparedness has emerged as a pivotal factor in digital learning success. Students with high digital readiness report significantly higher satisfaction and lower stress levels (Susanto and Najib, 2025). Yet surveys in Saudi Arabia reveal that university students demonstrate insufficient digital competencies, particularly in areas such as online collaboration, digital research, and cybersecurity awareness (Al-Motrif et al., 2025b).

At the institutional level, orchestration the strategic coordination of digital policies, infrastructure, training, and pedagogical support plays a critical role in shaping the digital learning ecosystem. Higher education institutions that demonstrate strong orchestration capabilities achieve up to 50% higher student satisfaction and more consistent learning outcomes (Ikram et al., 2025). In Saudi Arabia, institutional orchestration varies widely, with some higher education institutions offering digital support centers and others relying on fragmented or unsystematic approaches (Lee and Kim, 2025; Mahmood et al., 2025).

Moreover, learning well-being has emerged as a critical mediator construct in digital education research. It encompasses students’ emotional stability, sense of belonging, cognitive load, and perceived support within digital environments. Studies show that well-being strongly predicts satisfaction, persistence, and academic success in online learning (Li and Chen, 2025). In the Middle Eastern context, cultural factors such as collectivism, social connectedness, and family expectations further shape students’ well-being in digital settings (Yang and Badri, 2026). These issues underscore the importance of examining well-being as a mediating mechanism.

Despite this insight, significant gaps remain especially in the Middle East. Few studies have simultaneously examined awareness, acceptance, readiness, and institutional orchestration within a unified framework. Given Saudi Arabia’s rapid digital transformation, there is a pressing need for empirical research that captures the complex interrelationships among these variables within Saudi higher education institutions.

Accordingly, this study addresses these gaps by investigating how digital learning awareness, acceptance, readiness, and institutional orchestration influence student learning satisfaction through learning well-being in Saudi higher education institutions. Situating the research within the unique socio-cultural and technological context of Saudi Arabia, the study provides timely insights that can inform policy, strengthen institutional strategies, and support the Kingdom’s broader educational transformation. By integrating technological, institutional, and psychological dimensions within a unified framework, this study contributes to the growing body of research on sustainable digital transformation in higher education.

## 2 Review of related literature

### 2.1 Theoretical foundations

Understanding how Digital Learning Awareness (DLA), Digital Learning Acceptance (DLAc), Digital Learning Readiness (DLR), and Digital Learning Orchestration (DLO) shape Student Learning Well-Being (SLW) and ultimately Student Learning Satisfaction (SLS) requires a theoretical foundation capable of capturing the cognitive, behavioral, institutional, and psychological dimensions of digital learning.

The Technology Acceptance Model (TAM) provides the first pillar of this foundation. TAM contends that perceived usefulness and perceived ease of use determine users’ attitudes and behavioral intentions toward technology (Davis, 1989). These constructs have consistently predicted students’ engagement with learning management systems, virtual classrooms, and digital assessment tools in higher education (Orim et al., 2025). As Digital Learning Acceptance (DLAc) reflects students’ perceptions of usefulness and ease of use, TAM directly supports the role of acceptance as a key antecedent influencing both SLW and SLS. In the Saudi context, where rapid digital transformation under Vision 2030 has accelerated the adoption of advanced educational technologies, TAM is particularly relevant. Thus, TAM provides a robust theoretical justification for examining DLA as a determinant of SLW and SLS in Saudi higher education institutions.

While TAM explains individual acceptance, the Unified Theory of Acceptance and Use of Technology (UTAUT) extends this understanding by incorporating social influence, facilitating conditions, and performance expectancy (Halil and Ahmad, 2025). These constructs are especially pertinent in collectivist societies such as Saudi Arabia, where instructor encouragement, peer norms, and institutional authority significantly shape technology adoption behaviors (Hussein and Hassan, 2018). UTAUT therefore strengthens the theoretical grounding for Digital Learning Awareness (DLA) and Digital Learning Acceptance (DLAc), as both are influenced by social and institutional dynamics. Facilitating conditions such as reliable internet access, technical support, and structured training are also critical determinants of engagement and satisfaction in Saudi higher education institutions (Alruthaya, 2025). As these conditions directly influence students’ emotional comfort and cognitive load, UTAUT provides a strong rationale for linking DLA and DLAc to SLW and SLS. The theory’s emphasis on institutional support also aligns with Digital Learning Orchestration (DLO), reinforcing its relevance in shaping students’ digital learning experiences.

Digital Readiness Theory adds a complementary perspective by emphasizing students’ preparedness technological, psychological, and self-regulatory for digital learning (Alanoglu et al., 2025) Digital Learning Readiness (DLR) encompasses competencies such as device proficiency, online communication skills, time management, and the ability to self-direct learning. Empirical evidence demonstrates that students with higher readiness experience, lower cognitive load, reduced anxiety, and greater satisfaction with online learning (Gupta and Prashar, 2025). In Saudi Arabia, however, readiness remains uneven across regions and demographic groups, with nearly 35% of students lacking essential digital competencies (Alanoglu et al., 2025). Digital Readiness Theory therefore provides a strong justification for examining DLR as a predictor of both SLW and SLS in the Saudi context.

Institutional Theory provides the structural and institutional lens needed to understand Digital Learning Orchestration (DLO). Institutional theory asserts that institutional structures, norms, and governance systems shape individual behavior and perceptions (Galleli and Amaral, 2026). In digital learning, orchestration refers to the coordinated alignment of digital infrastructure, policies, training, support services, and pedagogical practices. In Saudi Arabia, institutional orchestration varies widely across higher education institutions, with leading institutions offering digital learning centers and 24/7 support. Given the central role of institutional trust in Saudi culture, DLO emerges as a critical determinant of SLW and SLS. Institutional Theory therefore provides a compelling justification for including DLO as a structural antecedent influencing students’ well-being and satisfaction.

Together, TAM, UTAUT, digital readiness theory, institutional theory, and well being frameworks form an integrated foundation that explain how awareness, acceptance, readiness, and orchestration affect satisfaction through well-being.

## 3 Thematic review of key constructs

**Figure 1:**
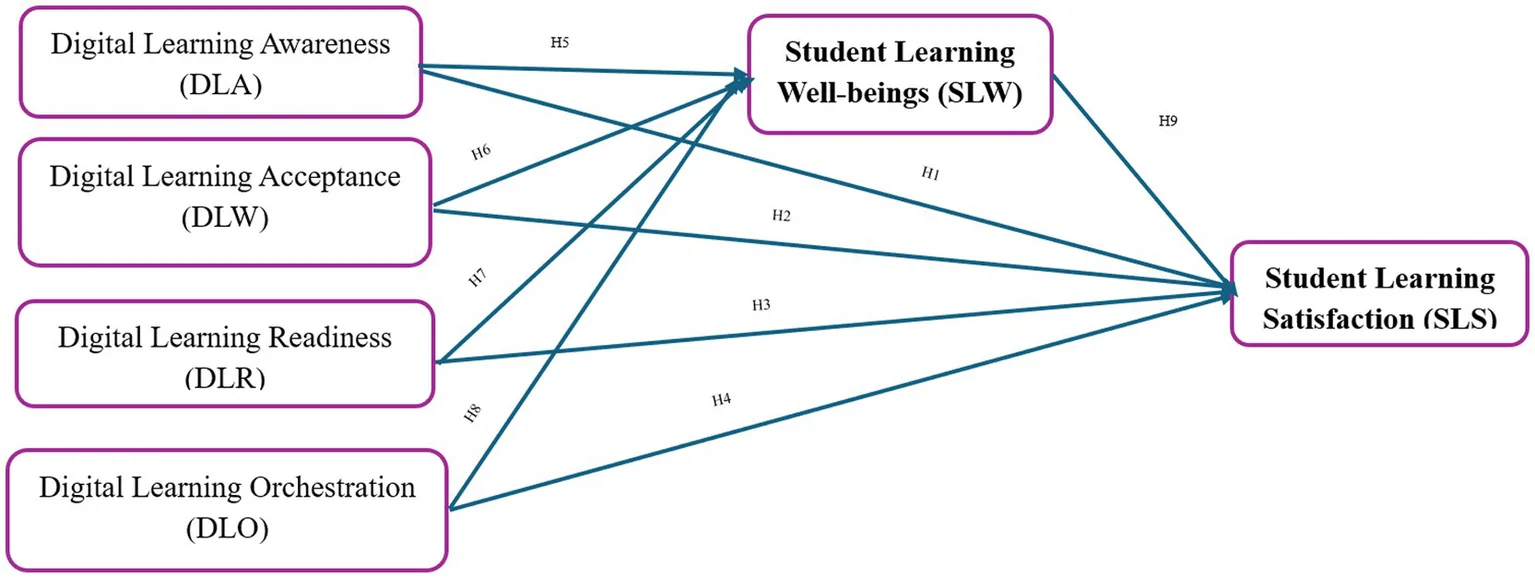
Conceptual framework. Source: (Mashfufah et al., 2019; Shaikh et al., 2026; Zhao and Feng, 2024).

The conceptual model (Figure 1) guiding this study illustrates a pathway through which the core dimensions of digital learning Digital Learning Awareness (DLA), Digital Learning Acceptance (DLAc), Digital Learning Readiness (DLR), and Digital Learning Orchestration (DLO) collectively shape students’ experiences in Saudi higher education institutions. These four constructs function as the primary antecedents that feed directly into Student Learning Well-Being (SLW), the central mediating mechanism in the model. By positioning SLW, the model emphasizes that students’ emotional stability, cognitive comfort, and sense of support are not incidental outcomes but essential psychological channels through which digital learning environments exert their influence. The final link from SLW to Student Learning Satisfaction (SLS) underscores that satisfaction emerges not merely from exposure to digital tools or institutional systems, but from the degree to which these elements foster a positive, healthy, and conducive learning experience. The framework further reflects the growing emphasis on learner-centered education, where students’ emotional, cognitive, and social experiences are recognized as essential components of effective digital pedagogy.

### 3.1 Digital learning awareness

Digital learning awareness refers to students’ understanding of the digital tools, platforms, pedagogical expectations, and learning processes embedded within online and blended learning environments (Gracıous and Shyla, 2012). Awareness functions as a cognitive antecedent to effective digital engagement, shaping how students interpret, navigate, and utilize digital learning resources. Empirical studies have demonstrated that students with higher awareness of digital learning systems exhibit stronger engagement, reduced uncertainty, and improved academic performance, with some reporting up to 30% higher learning effectiveness when awareness is high (Kim et al., 2019).

In Saudi Arabia, awareness levels vary considerably across institutions, reflecting disparities in digital literacy programs, orientation practices, and exposure to technology. Students in well-resourced higher education institutions particularly in Riyadh, Jeddah, and Dammam tend to exhibit higher awareness owing to structured digital onboarding and greater access to institutional support (Alayed, 2023). Conversely, students in newly established higher education institutions often report limited familiarity with digital platforms, which can undermine their confidence and emotional well-being. Given these disparities, it is reasonable to expect that higher digital learning awareness enhances students’ learning well-being and satisfaction.

Accordingly, the study postulates that higher digital learning awareness will positively influence learning well-being and, consequently, student learning satisfaction (H1).

### 3.2 Digital learning acceptance

Digital learning acceptance is a central determinant of students’ willingness to engage with online learning systems. Grounded in TAM and UTAUT, acceptance is shaped by perceived usefulness, perceived ease of use, social influence, and facilitating conditions (Alayed, 2023; Bayaga and du Plessis, 2024; Davis, 1989; Gonzalez-Tamayo et al., 2024; VanDeWiele et al., 2023). Research consistently demonstrates that acceptance predicts intention to use, depth of engagement, and satisfaction with digital learning environments. In the GCC region, however, acceptance is often moderated by concerns about interaction quality, assessment fairness, and platform reliability, with more than 40% of students expressing reservations toward fully online learning (Bensaid and Brahimi, 2020).

In Saudi Arabia, acceptance is strongly influenced by cultural expectations regarding instructor presence, trust in institutional systems, and prior exposure to technology. Students who perceive digital learning as aligned with their academic and cultural expectations are more likely to accept it and report positive learning experiences. Taken together, these dynamics suggest that acceptance is expected to be a strong predictor of both learning well-being and satisfaction.

Therefore, the study hypothesizes that digital learning acceptance will exert a positive effect on learning well-being and student learning satisfaction (H2).

### 3.3 Digital learning readiness

Digital readiness encompasses students’ technical competencies, self-regulation skills, time-management abilities, and psychological preparedness for digital learning. Research shows that students with high readiness experience lower cognitive load, reduced anxiety, and greater satisfaction with online learning (Fuchs et al., 2022). As such, readiness constitutes a foundational determinant of learning well-being.

In Saudi Arabia, readiness deficits remain a significant barrier to digital learning success. Studies indicate that nearly 35% of Saudi university students lack essential digital competencies, particularly in rural regions where access to technology is limited (Al-Motrif et al., 2025a). Gender differences have also been identified, with female students often reporting higher self-regulation but lower technical confidence (Abdulhay and Ahmadian, 2025). These patterns underscore the importance of institutional training and support programs. Given that readiness shapes both cognitive and emotional dimensions of learning, it is anticipated to significantly influence learning well-being and satisfaction.

Thus, the study hypothesizes that digital learning readiness will positively influence learning well-being and student learning satisfaction (H3).

### 3.4 Institutional orchestration

Institutional orchestration refers to the coordinated alignment of digital infrastructure, governance policies, faculty training, technical support, and pedagogical practices (Lee et al., 2026). Higher education institutions with strong orchestration achieve up to 50% higher student satisfaction attributed to improved system reliability, clearer expectations, and more consistent learning experiences (Rydzak et al., 2025).

In Saudi Arabia, institutional orchestration varies widely across higher education institutions (Khorsheed, 2015). Leading institutions have invested heavily in digital learning centers, faculty development programs, and 24/7 technical support. Given this variability, orchestration emerges as a critical determinant of students’ emotional stability, confidence, and well-being(Alruwaili et al., 2025).

Based on this evidence, the study hypothesizes that institutional orchestration will positively influence learning well-being and student learning satisfaction (H4).

### 3.5 Learning well-being

Learning well-being encompasses emotional, cognitive, and social dimensions of students’ experiences in digital learning environments. High well-being is associated with lower stress, greeter students’ engagement, and higher students’ satisfaction (Alruwaili et al., 2025). Well-being is influenced by platform usability, instructor support, peer interaction, and assessment fairness.

In Saudi Arabia, digital learning has introduced new well-being challenges. Students report increased stress related to online assessments, reduced peer interaction, and difficulties balancing domestic responsibilities with online coursework (Alruwaili et al., 2025).

Therefore, the study hypothesizes that learning well-being will positively influence student learning satisfaction and mediate the effects of awareness, acceptance, readiness, and orchestration (H5).

### 3.6 Student learning satisfaction

Student learning satisfaction reflects students’ appraisal of their digital learning experience (Bouranta et al., 2025). The educational context of Saudi higher education also provides an opportunity to examine how digitally mediated teaching and learning practices influence students’ academic engagement and perceived educational quality. Navarro et al. (2019) consistently show that satisfaction is a key predictor of academic persistence, engagement, and long-term learning outcomes. In Saudi Arabia, satisfaction levels vary significantly across higher education institutions, disciplines, and gender groups, reflecting disparities in digital infrastructure and institutional support (Mutawa, 2025; Parahoo et al., 2013).

Satisfaction also serves as a key outcome variable in digital learning research, capturing the cumulative effects of technological, psychological, and institutional factors. Given its centrality, it is imperative to examine how awareness, acceptance, readiness, orchestration, and well-being collectively determine this outcome.

Thus, the study hypothesizes that learning well-being will serve as a significant predictor of student learning satisfaction (H6).

## 4 Methodology

This study adopts a quantitative research design to empirically examine how Digital Learning Awareness (DLA), Digital Learning Acceptance (DLAc), Digital Learning Readiness (DLR), and Digital Learning Orchestration (DLO) influence Student Learning Well-Being (SLW) and Student Learning Satisfaction (SLS) among students across higher education institutions in the Hail region of Saudi Arabia. A quantitative approach is particularly appropriate for this study as it enables the systematic testing of theoretically grounded relationships and the measurement of latent constructs that characterize students’ digital learning experiences (Creswell and Creswell, 2017). Higher education institutions in the Hail region provide an ideal context for this study given its rapid digital transformation under Vision 2030 and its diverse student population, which reflects the broader demographic and technological realities of Saudi higher education (Allhidan, 2023; Shamrokh et al., 2022; Thomran and Alshammari, 2023).

Data were collected using an online questionnaire, a method widely recognized for its efficiency, accessibility, and suitability for digitally engaged populations. Online surveys were employed because they are efficient, accessible, and suitable for digital engaged student’ population. Moreover, as the study focuses on digital learning constructs, collecting data through a digital medium ensured methodological correlation between the research topic and the data collection process, thereby enhancing validity (Subramanian et al., 2023).

A combination of convenience sampling and snowball sampling was employed for data collection. Convenience sampling is widely used in higher education research owing to its practicality and ability to capture student experiences within natural academic settings (Kivunja, 2015). Across higher education institutions in Hail region, where students are dispersed across multiple colleges and campuses, convenience sampling enabled efficient access to a broad pool of respondents. To complement this approach, snowball sampling was incorporated, allowing initial participants to share the survey link with peers across different faculties and academic levels. Snowballing is particularly effective in collectivist cultures such as Saudi Arabia, where peer networks play a central role in information sharing and participation decisions (Almehmadi et al., 2014).

The survey was distributed primarily through WhatsApp, a widely used communication platform among higher education students in Saudi Arabia (Qiqieh et al., 2025; Alshammari et al., 2024). Research shows that social-media-based survey distribution enhances response rates, reduces sampling bias, and improves engagement among younger populations (Qiqieh et al., 2025). In the context of the Hail region, where digital communication is pervasive, WhatsApp distribution was both culturally appropriate and methodologically advantageous.

The questionnaire items were adapted from validated scales in digital learning, technology acceptance, readiness, orchestration, well-being, and satisfaction research. Items were modified to ensure cultural relevance and conceptual clarity for Saudi learners while maintaining theoretical rigor. All constructs were measured using a five-point Likert scale. Participation was restricted to students aged 18 and above who were actively engaged in digital learning across higher education institutions in the Hail region. Prior to completing the survey, participants provided electronic informed consent after reviewing information about the study’s purpose, confidentiality, and voluntary nature. Data collection was conducted over a two-month period from February to March 2026, coinciding with the second semester of the 2025–2026 academic year at the participating higher education institutions in the Hail region. Ethical approval was obtained from the relevant institution’s research ethics committee within the Hail region, ensuring adherence to institutional and national research standards.

Structural Equation Modeling using Partial Least Squares (PLS-SEM) was employed to analyze the data. PLS-SEM is particularly suitable for studies involving complex models with multiple latent constructs and mediating relationships (Changalima and Chuwa, 2025; Hair et al., 2025; Rosenzweig et al., 2025). It is also robust with medium-sized samples and non-normal data distributions, making it ideal for social science research conducted in real-world educational settings (Hair et al., 2025). This technique enables the simultaneous evaluation of measurement reliability, construct validity, and structural relationships, offering an assessment of the hypothesized framework. Thus, this study adhered to established reporting standards for quantitative research and PLS-SEM analysis to ensure methodological rigor and transparency.

## 5 Data analysis

The data analysis phase evaluates all research relationships embedded within the conceptual framework to derive meaningful findings from the survey data. The data were analysed using PLS-SEM to evaluate the measurement model and test the relationships between digital learning factors, student well-being, and learning satisfaction.

The data in Table 1 show a student profile from higher education institutions in the Hail region participating in the study, which demonstrates a favorable student composition in Saudi Arabia’s developing higher education system. The gender distribution shows 55.2% male students and 44.8% female students, which indicates Saudi Arabia continues to work toward achieving gender equality in digital learning accessibility.

**Table 1:** Demographical analysis.

S/n	Constructs	Scale	Frequency	Percent
1	Gender	Male	117	55.2
		Female	95	44.8
		Total	212	100
2	Age	18–20 Years	88	41.5
		21–22 Years	71	33.5
		23–25 Years	47	22.2
		26–28 Years	5	2.4
		29 + Years	1	0.5
		Total	212	100
3	Academic Level / Year of Study	First year	56	26.4
		Second year	39	18.4
		Third year	89	42
		Fourth year	25	11.8
		Postgraduate	3	1.4
		Total	212	100
4	Prior experience with online/digital learning	More than 3 online courses	164	77.4
		2–3 online courses	45	21.2
		One online course	2	0.9
		No prior experience	1	0.5
		Total	212	100

The study sample is predominantly young adults (18–22 years), with 77% having prior online course experience, indicating effective post-pandemic digital integration across higher institutions in Hail region.

### 5.1 Scales measurement

The evaluation of measurement instruments for Digital Learning Awareness (DLA), Digital Learning Acceptance (DLAc), Digital Learning Readiness (DLR), Digital Learning Orchestration (DLO), Student Learning Well-being (SLW) and Student Learning Satisfaction (SLS) is presented in the following section. The scales demonstrate high reliability and validity (Table 2). The composite reliability (CR) values exceeded 0.70 and average variance extracted (AVE) values surpased 0.50, thereby confirming convergent validity (Changalima and Chuwa, 2025; Hair et al., 2025). The items exhibit robust factor loadings, which exceed 0.80, confirming the reliability of the measurement model. Notably, the items DLA1, DLAc6, DLO5, and SLS6 items marked as “deleted,” showed evidence of preliminary assessment results which detected both low item loadings and cross-loading patterns. This suggests possible cultural or contextual differences in item wording and interpretation within the Saudi higher education context.

**Table 2:** Construct validity and reliability.

S/n	Constructs	Items	FA	CA	CR (rho_a)	AVE
1	Digital learning awareness (DLA)	DLA2	0.847	0.897696	0.8994175	0.710021
		DLA3	0.879			
		DLA4	0.824			
		DLA5	0.85			
		DLA6	0.813			
2	Digital learning acceptance (DLAc)	DLAc1	0.823	0.859238	0.8619955	0.641089
		DLAc2	0.793			
		DLAc3	0.826			
		DLAc4	0.834			
		DLAc5	0.722			
3	Digital learning readiness (DLR)	DLR1	0.777	0.889038	0.8913779	0.643611
		DLR2	0.817			
		DLR3	0.758			
		DLR4	0.813			
		DLR5	0.837			
		DLR6	0.809			
4	Digital learning orchestration (DLO)	DLO1	0.749	0.882277	0.892824	0.681652
		DLO2	0.853			
		DLO3	0.771			
		DLO4	0.892			
		DLO6	0.854			
5	Student learning well-being (SLW)	SLW1	0.766	0.902924	0.9053671	0.674081
		SLW2	0.803			
		SLW3	0.853			
		SLW4	0.853			
		SLW5	0.847			
		SLW6	0.8			
6	Student learning satisfaction (SLS)	SLS1	0.865	0.905943	0.9120482	0.726683
		SLS2	0.863			
		SLS3	0.814			
		SLS4	0.896			
		SLS5	0.821			

CA, Cronbach’s alpha; CR, Composite reliability (rho_a); AVE, Average variance extracted.

As shown in Table 2, Cronbach’s alpha (CA) values for all constructs are well above the accepted threshold of 0.70, ranging from 0.859 for Digital Learning Acceptance (DLAc) to 0.905 for Student Learning Satisfaction (SLS). The high CA values (CA > 0.80) reflect strong internal consistency as each construct contains items which effectively measure its core concept (Nunnally and Bernstein, 1994).

Furthermore, the Average Variance Extracted (AVE) scores, ranging from 0.641 (Digital Learning Acceptance) to 0.726 (Student Learning Satisfaction), establish the measures’ convergent validity. Specifically, the AVE values demonstrate that each construct accounts for more than 64% of the variance, which exceeds the 0.50 threshold recommended by Fornell and Larcker (1981). Taken together, these results indicate that the constructs achieve precise measurement while minimizing measurement errors, which is particularly critical given that digital learning constructs like readiness and acceptance and well-being present complex multidimensional characteristics.

Thus, the results analysis demonstrated satisfactory reliability and convergent validity for each construct, confirming the suitability of the measurement model for subsequent structural analysis (Hair et al., 2025; Bhatti et al., 2022).

### 5.2 Validity of measurements (convergent validity)

As presented in Table 3, the square root of the AVE for each construct exceeds its correlations with other constructs, which confirms adequate discriminant validity and demonstrates that the measures effectively capture conceptually distinct digital learning concepts.

**Table 3:** Discriminant validity of the total sample (Fronell–Larcker criterion).

Items	DLA	DLAc	DLR	DLO	SLW	SLS
DLA	0.842627					
DLAc	0.767783	0.800681				
DLR	0.666558	0.783155	0.802254			
DLO	0.699463	0.733522	0.825953	0.825622		
SLW	0.736929	0.829247	0.823852	0.832664	0.821025	
SLS	0.657256	0.744694	0.783186	0.744694	0.838504	0.852457

DLA, Digital learning awareness; DLAc, Digital learning acceptance; DLR, Digital learning readiness; DLO, Digital learning orchestration, SLW, Student learning well-being; SLS, Student learning satisfaction.

Further supporting construct validity, high AVE values, recorded above 0.64 for all constructs, indicate that more than 64% of item variance originates from the latent construct (Cheung et al., 2024; Fornell and Larcker, 1981). The analysis confirms that each construct contains items, which demonstrate sufficient correlation to measure their corresponding concepts, including Digital Learning Readiness and Student Well-Being. Importantly, these findings demonstrate high convergent validity, which is consistent with previous studies showing that digital education research requires exact measurement methods when examining specific cultural settings such as Saudi Arabia (Luppicini and Walabe, 2021), thereby rendering the study results both reliable and transferable to comparable contexts.

Turning to discriminant validity, the Fornell-Larcker criterion applied in Table 3 provides robust evidence through this established method, confirming that the measurement model constructs maintain empirically distinct relationships (Fornell and Larcker, 1981). Specifically, the square root of Average Variance Extracted (AVE) values for each construct exceeds their correlation with other constructs. For instance, Digital Learning Awareness (DLA) records an AVE of 0.842 and Digital Learning Acceptance (DLAc) records an AVE of 0.829 which exceed the correlation values of 0.666 between DLA and DLR and 0.699 between DLA and DLO. Collectively, these results confirm the distinct dimensions of digital learning experiences. Although the correlation between SLW and SLS was relatively high, the square root of AVE values remained higher than the inter-construct correlations, thereby satisfying the Fornell–Larcker criterion.

This study further demonstrates that the studied constructs including DLA and Student Learning Satisfaction (SLS) show strong discrimination among them, thereby reducing the risk of model integrity compromises arising from redundant or correlated variables. Notably, the AVE values remain stable at 0.726 for SLS and 0.674 for SLW throughout the analysis, confirming that the constructs preserve their theoretical and measurement distinctions (Cheung et al., 2024).

Moreover, the high correlations, such as 0.852 between SLW and SLS, while indicating strong relatedness as would be expected within an integrated model, still maintain sufficient discriminant validity, given that the AVE-based square root values are higher than these inter-construct correlations. As such, the research results fulfill the standard established by Fornell and Larcker (1981). In sum, the results presented in Table 3 confirm that the constructs operate independently in examining digital learning dynamics, as each captures a conceptually distinct aspect of the phenomenon, thus reinforcing the reliability of the subsequent structural analyses.

### 5.3 Model measurement index

**Figure 2:**
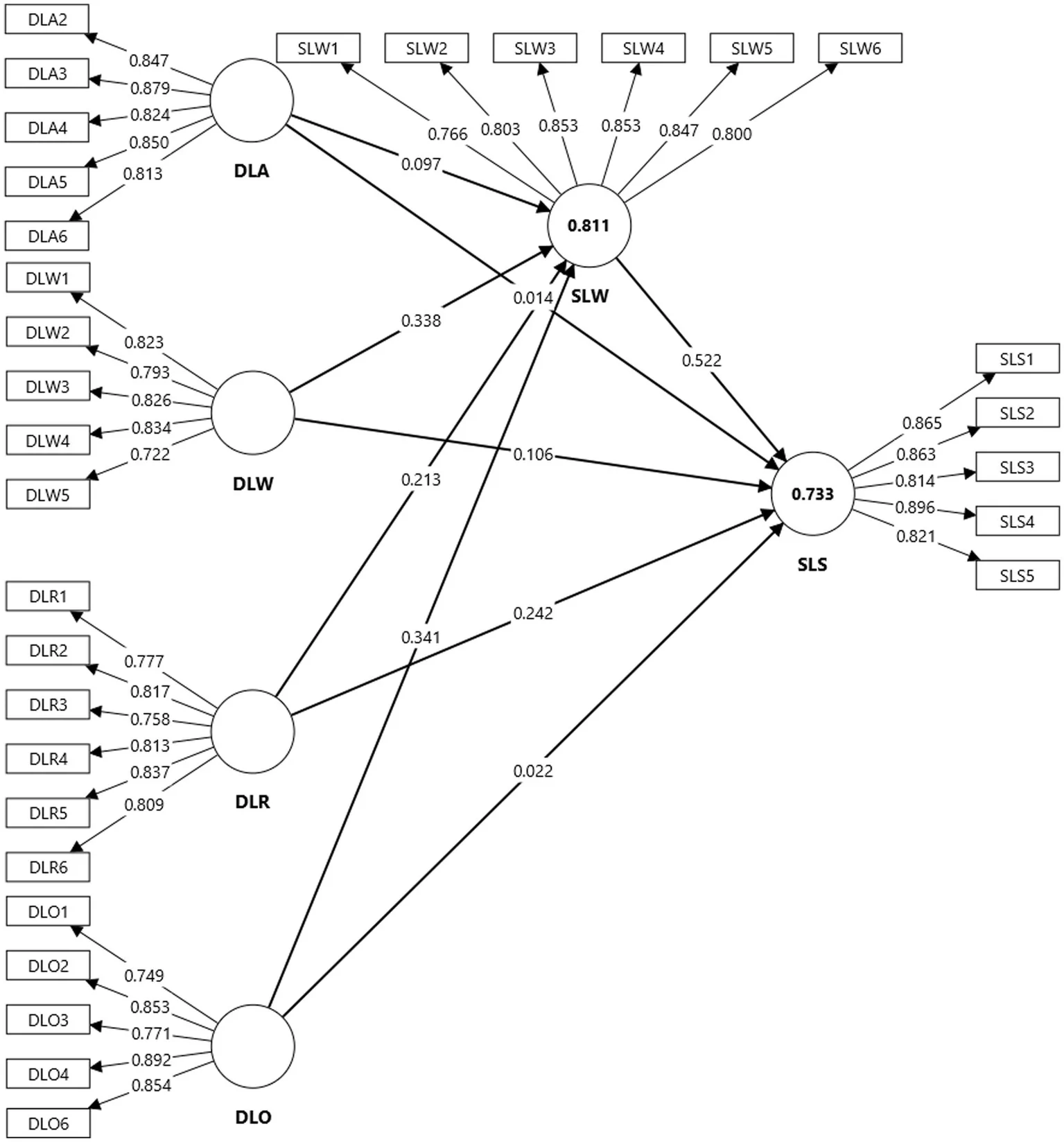
Measurement model. DLA, Digital learning awareness; DLAc, Digital learning acceptance; DLR, Digital learning readiness; DLO, Digital learning orchestration; SLW, Student learning well-being; SLS, Student learning satisfaction.

As shown in the Figure 2, all measured variables (indicators) load strongly onto their corresponding latent constructs, which include DLA, DLAc, DLR, DLO, SLW, and SLS with all factor loadings exceeding 0.70 (Hair et al., 2025). The Composite Reliability (CR) values confirm high reliability exceeding 0.70 for all constructs including SLW at 0.902 and SLS at 0.912.

The Average Variance Extracted (AVE) values ranging from 0.641 to 0.726 confirm that the research constructs have established strong convergent validity. In particular, the AVE value of 0.726 for SLS indicates that its indicators account for a significant proportion of variance, supporting the notion that its measurement items demonstrate strong correlation and effectively represent the construct.

As illustrated in Figure 2, the measurement model demonstrates effective digital learning assessment through its high loadings and reliable indices, which measure digital learning dimensions of awareness and acceptance, readiness, orchestration, well-being and satisfaction. Cheung et al. (2024) and Hair et al. (2025) emphasize that researchers need to develop a well-specified measurement model to accurately interpret their structural model paths.

To ensure the validity and robustness of the structural model, comprehensive validation procedures were followed. The high R-squared values of 0.733 for Student Learning Satisfaction and 0.811 for Student Learning Well-being indicate strong explanatory power. These relationships were confirmed through rigorous measures including reliable measurement models with Cronbach’s alpha values above 0.86 and AVE exceeding 0.64, establishing strong convergent validity. Discriminant validity was verified using the Fornell-Larcker criterion, ensuring the constructs are conceptually distinct. Additionally, checks for multicollinearity through Variance Inflation Factor (VIF) values below 5.0, alongside bootstrapping validation, helped confirm that the findings are not the result of common method bias or methodological artifacts, providing confidence in the model’s empirical robustness.

The R-squared values for Student Learning Satisfaction (SLS) and Students Learning Well-Being (SLW) as endogenous latent variables appear in Table 4 with values of 0.733 and 0.811. These figures reflect the proportion of variance in these key outcome constructs accounted for by the model and are indicative of a highly predictive and well-specified model. The R-squared value of 0.733 for SLS indicates that the model explains 73.3% of student satisfaction with digital learning through its predictors, which include learning well-being and digital learning factor.

**Table 4:** Variance explain in the endogenous latent variable.

	R-square	R-square adjusted
SLS	0.733	0.727
SLW	0.811	0.807

SLW, Student learning well-being; SLS, Student learning satisfaction.

Research in behavioural and educational fields recognised this figure as an indicative of strong predictive ability while providing a solid empirical basis for both theoretical and practical applications. Variance Inflation Factor (VIF) values for all predictor constructs were below the recommended threshold of 5.0, indicating that multicollinearity was not a significant concern. The R-squared value of 0.811 for SLW indicates that digital learning constructs which include awareness, acceptance, readiness and orchestration explain more than 81% of the total variance in learning well-being. The high R^2^ values indicate strong explanatory power and confirm the model’s predictive relevance for digital learning outcomes.

**Figure 3:**
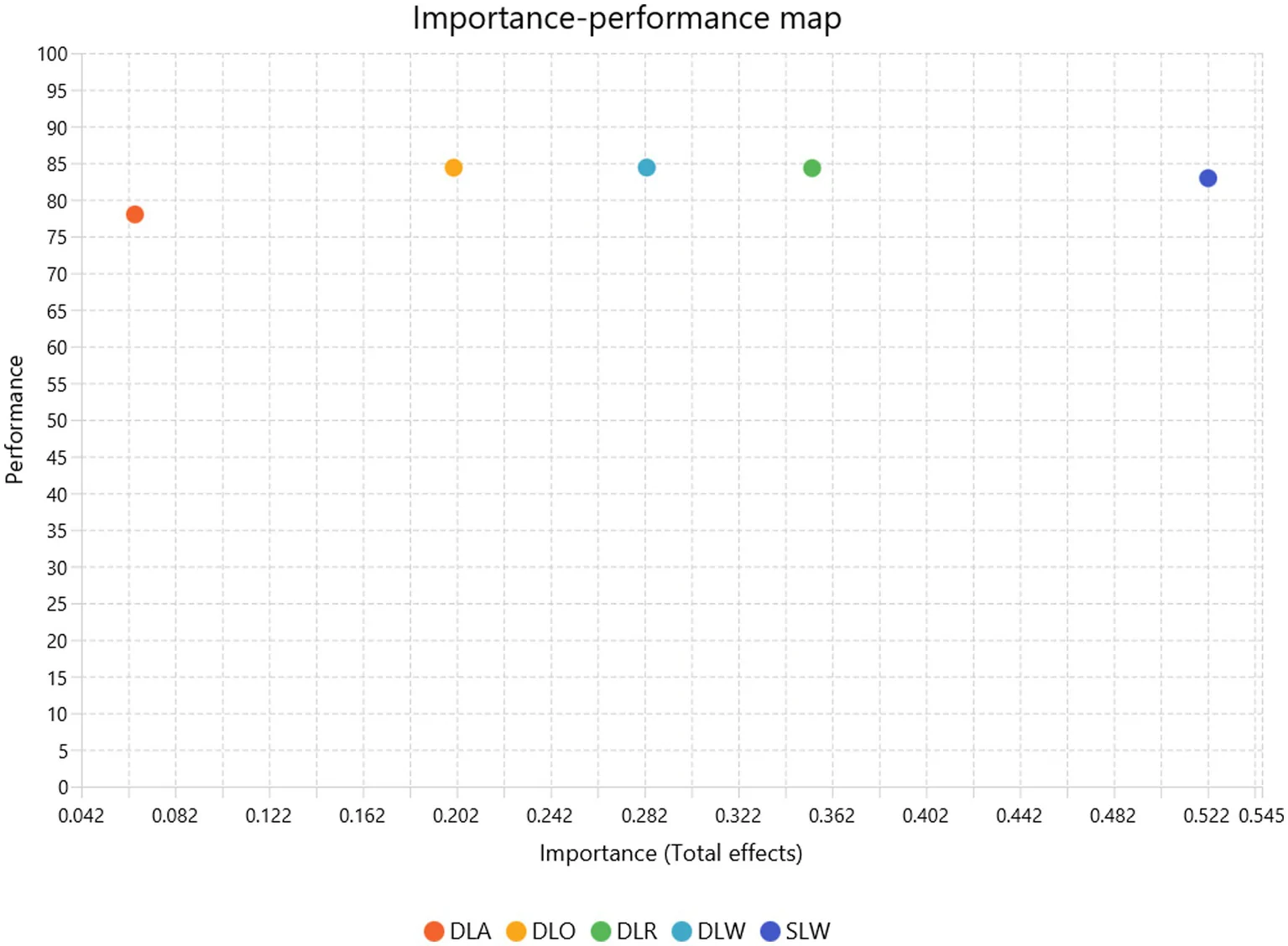
Importance performance map.

The Importance Performance Map (Figure 3) demonstrates how to enhance digital learning for students through its visual display, which presents vital elements together with their importance ratings and current performance metrics. According to the analysis, Student Learning Well-Being (SLW) and Digital Learning Readiness (DLR) significantly predict Student Learning Satisfaction (SLS) with standardized beta coefficients totaling 0.522 and 0.242, and their t-values reach 4.588 and 2.569 (Table 5, *p* < 0.01 and *p* < 0.05). These results indicate that students achieve higher satisfaction when educators focus on developing their emotional stability, cognitive balance and digital competencies.

**Table 5:** Hypotheses testing in structural model.

H	Relationship	Std. Beta	Std. Dev	t-values	*p* values	Findings
H1	DLA → SLS	0.014	0.088	0.159	0.874	Not Supported
H2	DLAc → SLS	0.106	0.087	1.229	0.220	Not Supported
H3	DLR → SLS	0.242	0.094	2.569	0.010	Supported
H4	DLO → SLS	0.022	0.115	0.194	0.847	Not Supported
H5	DLA → SLW → SLS	0.051	0.034	1.472	0.142	Not Supported
H6	DLAc → SLW → SLS	0.176	0.054	3.237	0.001	Supported
H7	DLR → SLW → SLS	0.111	0.045	2.446	0.015	Supported
H8	DLO → SLW → SLS	0.178	0.051	3.512	0.000	Supported
H9	SLW → SLS	0.522	0.114	4.588	0.000	Supported

DLA, Digital learning awareness; DLAc, Digital learning acceptance; DLR, Digital learning readiness; DLO, Digital learning orchestration; SLW, Student learning well-being; SLS, Student learning satisfaction.

The map also shows that Digital Learning Acceptance (DLW) and Digital Learning Awareness (DLA) hold similar levels of importance, yet their performance results differ from each other. The structural model shows that DLA does not have a direct impact on satisfaction or well-being as its path coefficients fail to reach statistical significance (H1 and H2, Table 5). This is consistent with research by Han et al. (2025), who found that students from higher education institutions who understand more about a topic will take part in extra activities. Similarly, Naseer et al. (2025) report up to 30% higher learning effectiveness among students with strong awareness, highlighting an opportunity for targeted interventions to strengthen this construct further.

The map identifies Digital Learning Orchestration (DLO) as a significant contributor to SLW and SLS, with a beta coefficient of 0.178 (*p* < 0.001). This suggests that orchestration plays a major role in shaping institutional support systems, including faculty development and technological infrastructure. The map shows how these variables converge to create an optimal SLW, which improves when institutions provide more support to students who already demonstrate digital tool readiness and acceptance. Given that SLW account for 81% of the variance in SLS (*R*^2^ = 0.733, Table 4), institutions should priorities these essential areas.

Singun (2025) and Surjawan et al. (2025) reported that digital higher education institutions achieve better student outcomes through investment in psychological support and digital competency training and effective coordination of institutional operations. Therefore, the research findings identify the specific improvements needed to develop a digital learning environment that enhance student learning experiences and engagement, ultimately contributing to an educational ecosystem centered on student needs.

**Figure 4:**
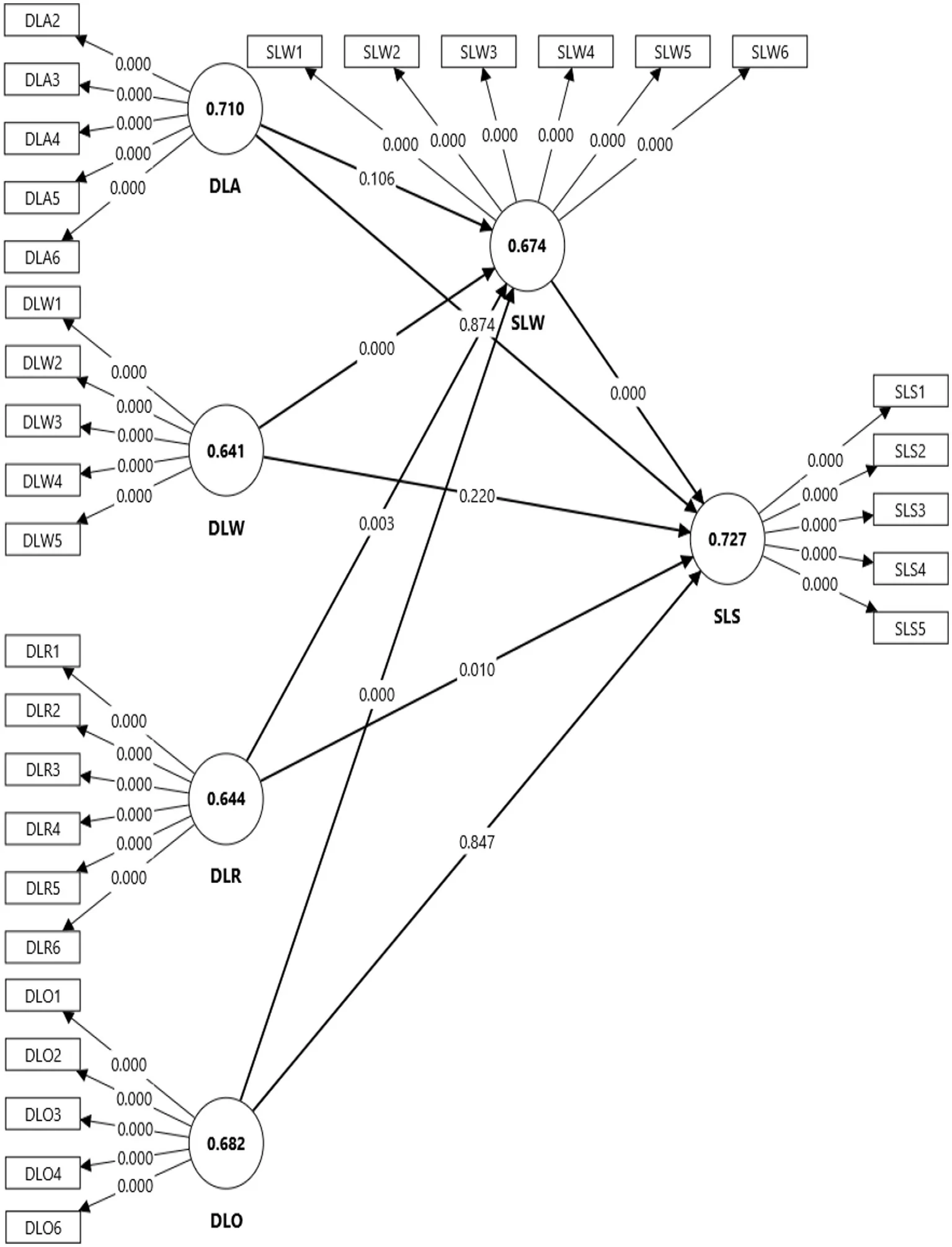
Bootstrapping model. DLA, Digital learning awareness; DLAc, Digital learning acceptance; DLR, Digital learning readiness; DLO, Digital learning orchestration; SLW, Student learning well-being; SLS, Student learning satisfaction.

The bootstrap analysis (Figure 4) shows that Student Learning Well-being (SLW) exerts the greatest effect on Student Learning Satisfaction (SLS), as it has a standardized coefficient of 0.522 (*p* < 0.001). The research shows that students will achieve higher digital learning satisfaction when institutions provide adequate support that helps them maintain emotional stability, fosters cognitive engagement, and nurtures social relationships. The research shows that Digital Learning Readiness (DLR) and Digital Learning Orchestration (DLO) positively contribute to SLW, with a beta value of 0.111 (*p* = 0.015) and 0.178 (*p* < 0.001) demonstrate that technological competencies, institutional support, and structured support systems are significant determinants. The research findings confirm the study of Weng and Wirda (2025), which demonstrated that student readiness, and higher education institutional support serve as essential factors in reducing student anxiety, promoting student participation and creating an enriching learning environment that leads to better academic achievement.

The research data shows that Digital Learning Acceptance (DLW) has a positive relationship with satisfaction (*β* = 0.106), although the results do not reach statistical significance at the conventional research threshold (*p* = 0.220). The results show that Digital Learning Awareness (DLA) does not exert any significant impact on satisfaction or well-being, as awareness alone does not yield better results when institutions lack acceptance, readiness, and adequate institutional support. Investing in these psychological and structural dimensions offers a practical pathway to elevating the quality of digital learning experiences, aligning with prior empirical and theoretical evidence.

### 5.4 Hypotheses testing in structural model

The hypothesis testing results demonstrate strong evidence that supports a digital learning initiative strategic shift toward well-being-focused programs.

Starting with the first hypothesis, H1 (DLA → SLS), the analysis shows no significant impact on satisfaction as the statistical analysis reveals no meaningful relationship (Beta = 0.014, *p* = 0.874). This implies that digital learning awareness does not produce any positive effects on students’ satisfaction outcomes. Research shows that awareness alone does not lead to better satisfaction results as it needs to be combined with appropriate support and positive emotional responses. Suduc et al. (2010), showed that user awareness leads to better system interaction as users find value in the system and as their environment supports system usage. Therefore, policy needs to link educational curricula with targeted programs which foster psychological well-being and active participation. Digital Learning Awareness (DLA) alone may not have a major impact on student satisfaction. Awareness is a primarily cognitive attribute and does not necessarily contribute to a positive emotional engagement or behavioral adoption of digital learning environments. Based on the Technology Acceptance Model (TAM) (Davis, 1989), perceptions of usefulness and ease of use are more influential factors that determine satisfaction than awareness alone. Likewise, the Unified Theory of Acceptance and Use of Technology (UTAUT) emphasises the significance of social influence and facilitating conditions to affect technology use behaviors in collectivist cultures that include Saudi Arabia (VanDeWiele et al., 2023; Bayaga and du Plessis, 2024; Gonzalez-Tamayo et al., 2024). In Saudi Arabia, cultural factors such as social connectedness and expectations from family members are more likely to influence attitudes towards digital learning than awareness itself (Luppicini and Walabe, 2021; Salisu et al., 2026). However, while students are generally familiar with digital tools, they may not possess the confidence, acceptance, or institutional support to leverage them in meaningful ways, which could diminish the effect of awareness on overall satisfaction. This implies that awareness should be combined with a perception of usefulness, acceptance, readiness and efficient institutional orchestration for a positive impact on educational experiences and satisfaction levels.

Similarly, the research findings failed to support H2 (DLW → SLS), as study results indicated that acceptance variables did not directly influence the results (Beta = 0.106, *p* = 0.220). While acceptance as often regarded as critical based on the Technology Acceptance Model (Davis, 1989), the findings suggest that in the context of Saudi higher education institutions, acceptance might influence satisfaction indirectly through mediating constructs such as well-being. The study by Al Hashlamoun (2021) reveals that GCC students face uncertainty about online learning owing to concerns regarding the absence of human interaction and inadequate assessment procedures.

In contrast, the results from H3 (DLR → SLS) indicate a statistically significant positive relationship (Beta = 0.242, *p* = 0.010), demonstrating that digital readiness constitutes an essential determinant. Rehman et al. (2024) show that students who possess strong digital competencies will experience decreased cognitive overload, which results in better academic achievement. Saudi Arabia needs to address its digital skills deficit, as 35% of its students lack basic digital competencies (Hamid et al., 2025), highlighting the need for targeted training programs to enhance digital literacy skills and improve student satisfaction, participation, and academic success in alignment with Vision 2030 objectives. The EU has shown that student satisfaction levels determine online learning persistence, as satisfied students demonstrated better retention rates (Bernardo et al., 2025; Okros et al., 2025).

While H4 (DLO → SLS) was not supported (Beta = 0.022, *p* = 0.847), the path coefficient was positive; the relationship was statistically negligible and provides no empirical support for a direct relationship between institutional orchestration and student satisfaction yet suggests potential when combined with other factors. Gautam et al. (2025) demonstrate that higher education institutions that establish digital infrastructure systems and pedagogical support achieve student satisfaction rates 50% above average. The non-significance results may be attributed to variability in implementation quality across Saudi institutions indicating that orchestration requires continuous and systematic integration rather than being treated as a separate initiative. These findings indicate that institutional orchestration may influence satisfaction indirectly through improvements in student well-being rather than through a direct pathway.

The study produced its most robust finding through H5 (SLW → SLS) with a Beta value of 0.522 (*p* < 0.001), confirming that learning well-being functions as a key mediator which affects student satisfaction. The research results from Zong and Yang (2025) demonstrate that students who learn to control their emotions and build social connections will stay interested in their studies and continue learning. The online testing environment in Saudi Arabia produces student stress that exceeds normal levels by 34.8%. Alghamdi et al. (2025) suggesting that virtual peer groups and stress management programs together with specific psychological and social support systems will enhance student satisfaction while decreasing student departure rates. Thus, the results show that digital education success depends on policies that create emotional safety and cognitive balance as their fundamental foundation.

The H6 (DLW → SLW) and H7 (DLR → SLW) yielded statistically significant positive results reached *p* = 0.001 and *p* = 0.015, respectively. The study shows students must accept digital tools before they can use them as their readiness for these tools produces improved well-being results (Alghamdi et al., 2025), while students who feel at ease with digital tools show 25% lower anxiety levels leading to better satisfaction results (Stefanovic and Klochkova, 2021). The evidence from H8 (DLO → SLW) shows that institutional orchestration leads to better well-being as research shows that students who receive organized educational support will drop out at lower rates of 15–20% (Bernardo et al., 2025). These findings indicate that institutional orchestration may influence satisfaction indirectly through improvements in student well-being rather than through a direct pathway.

## 6 Discussion

The current study investigated relationships between digital learning awareness, acceptance, readiness, orchestration and learning satisfaction among higher education students in Saudi Arabia and the wider Middle East. Using a conceptual framework based on the Technology Acceptance Model (TAM), Unified Theory of Acceptance and Use of Technology (UTAUT), Digital Readiness Theory, the Institutional Theory and the Well-Being Theory, results of the study supported both direct and indirect paths to students’ learning satisfaction. To strengthen the conceptual coherence of the study findings, it is important to clarify how each construct contributes to the broader digital learning ecosystem.

H1 (awareness) and H4 (orchestration → satisfaction) were not supported directly; H2 (acceptance) only indirectly via well-being; H3 (readiness) had the strongest direct effect on satisfaction. The four acceptance variables (H5–H8) testing the mediator effects of Student Learning were regressed upon the four awareness variables with satisfaction as the outcome variable. Student Learning Well-Being significantly mediated the other three acceptance–satisfaction relations (H6 through H8); however, it did not significantly mediate the awareness–satisfaction relation (H5: *β* = 0.051, *p* = 0.142). Contrary to an overall mediation pattern, awareness, acceptance, readiness, and orchestration all affect satisfaction of the learning experience through Student Learning Well-Being only when Well-Being served as a full intermediary for the three constructs. Student Learning Well-Being was the strongest positive predictor of students’ satisfaction with their digital learning experience (H9: β = 0.522, *p* < 0.001). This finding is important for the Saudi and Middle Eastern culture characterizes by collectivist values of social connectivity and of family taking care of children and students. In such contexts, a supportive digital environment can therefore contribute more to students’ positive experience in their digital learning environment (Yang and Badri, 2026).

In summary, student satisfaction with digital higher education is not directly caused by students’ knowledge of services provided by an institution, nor by the amount of money an institution invests in the digital teaching and learning environment. What matters most is how an institution supports students’ well-being and creates a cohesive digital ecosystem that supports learning. Students accept environments that support their readiness to engage with learning environments managed in a systematic and orchestrated manner rather than as separate projects or initiatives. Thus, digital education quality depends on institutional support, LMS quality, faculty competencies, and students’ well-being.

### 6.1 Practical implications

The outcomes of this study offer a clear road map for Saudi higher education institutions that aspire to establish digital learning environments in which students not only manage to survive but also thrive, a goal that lies at the foundation of Vision 2030’s ambitions for a digital transformation, academic excellence, and human development. The most important metric of student satisfaction and well-being identified in this study was digital learning readiness (H3). However, approximately 35% of students, predominantly from the Hail region, lack adequate digital skills. It is imperative that institutions design comprehensive digital literacy programs that include workshops, certification courses, and training on self-regulation, time management, and technological competence to ensure students can manage the cognitive load and reduce stress levels in the online environment (Ibrahim et al., 2025).

Similarly, digital learning orchestration (H4) calls for institutions to move beyond isolated infrastructure upgrades and establish a more unified, institution-wide alignment of high-speed connectivity, multi-platform integration, continuous system availability, and coordinated faculty training an approach that, when implemented effectively, has already been observed to boost student satisfaction rates by up to 50% (Singh et al., 2025). Since social–emotional learning initiatives have been consistently associated with a 30% decrease in dropout rates and a 15–20% enhancement in academic performance across the globe (Nichols, 2025), institutions are encouraged to invest in psychological support ecosystems peer interaction environments, stress management programs, and culturally aligned well-being workshops that honour Saudi collectivist values and family involvement, given that student educational well-being (SLW) mediates the essential paths from acceptance and orchestration to satisfaction (H6, H8, H9).

Consequently, it is imperative to implement data-driven decision-making to sustain these gains: institutions that incorporate a combination of learning analytics and continuous feedback mechanisms into their operations have demonstrated the ability to improve student retention by 10–12% while simultaneously increasing satisfaction (Almusfar, 2025). Moreover, those that innovate with blended learning models as King Saud University has demonstrated achieving 25% higher levels of student satisfaction over two consecutive years (Baksh et al., 2025), illustrated how innovation, institutional strategy, and human-centred values converge within a unified digital education vision.

### 6.2 Policy implications

Policy makers should mandate integrating a digital literacy framework, a unified infrastructure, and mental health support as co-equal pillars under Vision 2030. With 35% of higher education students exhibiting measurable gaps in digital competency, experienced most acutely in underserved communities, national funding needs to be directed towards establishing mandatory digital literacy programs that incorporate certification pathways along with industry partnerships, ensuring quality digital education is considered a right, not a privilege.

Singapore’s Ministry of Education has already tested and confirmed a model framework that resulted in a 15–20% reduction in students dropout rates through coherence national digital policy (Lee, 2025). As H5 posits students learning well-being (SLW) mediates between these technological and institutional investments and student satisfaction. Therefore, students’ mental health must be elevated from a peripheral concern to a policy priority, with national guidelines mandating the integration of virtual mental health services, specialized stress management workshops, and peer support networks within digital learning environments, so that every student, regardless of socioeconomic circumstances, has the psychological scaffolding necessary to achieve success.

### 6.3 Limitations of the study

This study has several limitations that should be acknowledged. First, the study focuses on higher education’s institutions in the Hail Region, which may limit the generalizability of the study findings across Saudi Arabia. Secondly, the use of a cross-sectional design captured students’ perceptions at a single point in time.

Culturally, the measuring instruments are stamped with the quiet impressions of Western educational assumptions, which have the potential to distort what is being measured in a context where, as Schwarz and Chin (2007) pointed out, constructs like acceptance and satisfaction mean fundamentally different things. Students may respond through lenses of institutional trust and social harmony rather than personal fulfilment, silently modifying the data in ways that may underestimate certain constructs.

Furthermore, the study’s limitations were further shaped by who participated and how the data were collected. Convenience and snowball sampling attracted significantly more digitally engaged students, while self-report questionnaires elicited the familiar triumvirate of social desirability, inadequate recall, and non-response bias, with the most preoccupied and dissatisfied students being quietly excluded from the data altogether. The research extends beyond sampling to look at awareness, acceptance, readiness and orchestration, but the study does not enter the pedagogical core, where outcomes are actually determined by conventional teaching techniques, gamification and personalized learning, the three components that Bontchev et al. (2021) and Hwang et al. (2012) acknowledge as key for student focus and mental health. While the use of convenience and snowball sampling through WhatsApp offers practical advantages in reaching out to a wide and digitally engaged student population in a timely manner, it also inevitably brings about some degree of bias in the selection process by disproportionately representing highly connected, digitally savvy college students (Kivunja, 2015). While this is a limitation, the study highlights the significance of transparent reporting of sample characteristics in order to ensure the outcomes can be taken into account in the framework of the sample’s digital engagement. Moreover, the research underscores its dedication to broad inclusivity and validity by supplementing this approach with focused outreach to underrepresented groups through alternative methods in future studies. This approach takes advantage of the widespread use of WhatsApp in Saudi Arabia, within which social media platforms are an integral part of student communication, and is mindful of the biases inherent in this method to ensure the robustness and significance of the findings in the constantly shifting digital education landscape (Kivunja, 2015). The construct of well-being (SLW), replete with emotional, social, and cognitive, requires measurement instruments that are far more culturally diverse and multi-dimensional than those readily accessible at the moment. At the heart of the issues is the relentless pace of technological change: as AI, blockchain and virtual reality are poised to transform learning environments in the next 2 to 3 years, today’s findings risk becoming yesterday’s map. Nonetheless, the study’s findings are genuinely instructive for Saudi institutions and emerging digital economies globally as they navigate the shared environment of student well-being, digital readiness, and educational transformation. Future studies should adopt longitudinal and mixed methods designs to capture evolving patterns of digital learning experiences across diverse institutional contexts.

### 6.4 Direction of future research

The research findings from this study will serve as fundamental knowledge for Saudi higher education digital learning so researchers can apply this knowledge to create new educational technologies which they should test within the local environment to develop improved learning methods. The initiatives will establish Saudi Arabia and similar areas as leaders who develop a digital academic environment which provide complete accessibility and strong resistance to challenges while supporting national development goals like Saudi Vision 2030.

The research yields vital findings about student technology awareness as it affects their willingness to adopt technology, their preparedness and their mental wellness. For example, a study in the USA demonstrated that sustained engagement with digital platforms correlated with a more than 30% increase in graduation rates over 5 years (Thelisdort, 2025). The research methods operating in Saudi Arabia enable scientists to monitor student achievement results which result from digital transformation across various time spans.

The success of digital learning requires proper functioning policy systems which need to work with educational institutions that possess enough capabilities. Research activities need to develop strategic institutional orchestration methods by building digital infrastructure, teaching faculty members and establishing student service programs. Yelamarthi et al. (2025) demonstrate that higher education institutions which create their own digital support centers obtain student satisfaction and retention rates between 55% and 60%. Hence, future studies should adopt longitudinal and mixed-method approaches to examine digital learning experiences across different Saudi regions and institutional contexts. Researchers should also investigate emerging technologies such as AI, VR, and adaptive learning systems within culturally relevant educational environments.

Given that, the current methods used to measure digital learning constructs stem from Western societies thus, researchers need to develop culturally appropriate assessment tools which will measure perceptions of Saudi and Middle Eastern populations accurately. The way students from higher education institutions view acceptance and well-being differs from individualistic societies, as they place more value on peer support and social connections (Alsharif et al., 2025). The reliance on Western-centric measurement scales for constructs such as acceptance, satisfaction, and well-being currently carries a significant risk of distorting the culturally specific values that define Saudi students’ educational experiences. These tools tend to emphasize personal achievement and individual gratification, overlooking the collectivist mindset, social harmony, family involvement, and community trust that characterize Middle Eastern cultures (Almehmadi et al., 2014). Such discrepancies can lead to inaccurate assessments, underappreciation of peer support, and masking of social interconnectedness, which can threaten the validity of research findings. Future studies should move beyond generic frameworks and develop indigenous-based and culturally grounded measurement tools that reflect the collectivist values in Saudi society. Qualitative methods, including interviews, focus groups, and ethnographies, can complement the quantitative methods by adding deep understanding to student’s lived experiences and cultural norms, which allows for a more complex understanding of quantitative data in an appropriate cultural context. This multimethod, culturally sensitive approach proves essential for developing valid and reliable assessments that accurately capture the educational and well-being experiences of Saudi students, instead of having to depend on potentially Western perspectives.

Future studies need to confirm the cultural appropriateness of assessment tools through mixed-methods research which combines qualitative data with quantitative statistical analysis to improve both measurement accuracy and result transferability. The creation of educational instruments which target particular learning environments improved accuracy for predicting student achievement and satisfaction levels.

## 7 Conclusion

The findings suggest that digital learning initiatives within Saudi higher education institutions are increasingly supporting students’ educational experiences, particularly through improvements in digital readiness and institutional support. Research findings demonstrate that digital learning readiness (DLR) and institutional orchestration (DLO) function as vital elements which boost student satisfaction and well-being as they affect student well-being which serves as a crucial connection between them. The study contributes theoretically by integrating multiple technology and well-being perspectives to explain students’ digital learning satisfaction within the context of Saudi Arabia.

Theoretically, the study integrates TAM, UTAUT, Digital Readiness, Institutional, and Well-Being theories, showing that acceptance, readiness, and orchestration operate through well-being to drive satisfaction not directly.

At the strategical level, the theoretical framework shows that higher education institutions need to create ongoing assessment systems which will enable them to develop adaptable policies through data evaluation and student feedback assessment. Institutions can create improved strategic plans through data-based methods which support their digital transformation process by implementing methods that achieve Vision 2030 targets for economic diversification and human capital development. Ultimately, the future of digital higher education depends not only on technological advancement, but also on institutions’ ability to create inclusive, supportive, and pedagogically meaningful learning environments that enhance students’ academic and emotional experiences. Sustainable digital education therefore requires a balanced integration of technological innovation, learner well-being, and educational quality enhancement.

## Data Availability

The raw data supporting the conclusions of this article will be made available by the authors, without undue reservation.
