# Correction: From digital learning readiness to student satisfaction: the mediating role of learning well-being in Saudi higher education

**DOI:** 10.3389/frai.2026.1978594

**Published:** 2026-09-08

**Authors:** Aliyu Alhaji Abubakar, Essa Mubrik N. Almutairi, Hilal Nafil Alhulail, Sayf Mohamed Dhiya Younis Al-Ashqar, Habiba Muhammad Abdullahi

**Affiliations:** 1 Department of Management Information System, College of Business, University of Ha'il, Ha'il, Saudi Arabia; 2 Humanities Research Centre, University of Ha'il, Ha'il, Saudi Arabia; 3 Department of Marketing, College of Business, University of Ha'il, Ha'il, Saudi Arabia; 4 Business Administration Department, College of Administration and Economics, University of Mosul, Mosul, Iraq; 5 Department of Entrepreneurship & Innovation Studies, Federal University Kashere, Kashere, Nigeria

**Keywords:** digital education, digital learning awareness, higher education institutions, learning well-being, Saudi Arabia, student satisfaction

Affiliation Humanities Research Centre, University of Ha'il, Ha'il, Saudi Arabia was omitted for author(s) Aliyu Alhaji Abubakar, Essa Mubrik N. Almutairi, and Hilal Nafil Alhulail. This affiliation has now been added for author(s) Aliyu Alhaji Abubakar, Essa Mubrik N. Almutairi, and Hilal Nafil Alhulail.

The original version of this article has been updated.

